# Placental abruption and long-term maternal cardiovascular disease mortality: a population-based registry study in Norway and Sweden

**DOI:** 10.1007/s10654-015-0067-9

**Published:** 2015-07-16

**Authors:** Lisa DeRoo, Rolv Skjærven, Allen Wilcox, Kari Klungsøyr, Anna-Karin Wikström, Nils-Halvdan Morken, Sven Cnattingius

**Affiliations:** Department of Global Public Health and Primary Health Care, University of Bergen, Postboks 7804, 5018 Bergen, Norway; Medical Birth Registry of Norway, Norwegian Institute of Public Health, Bergen, Norway; National Institute of Environmental Health Sciences, National Institutes of Health, Research Triangle Park, NC USA; Department of Women’s and Children’s Health, Uppsala University, Uppsala, Sweden; Department of Clinical Sciences, University of Bergen, Bergen, Norway; Clinical Epidemiology Unit, Department of Medicine, Karolinska University Hospital and Karolinska Institute, Stockholm, Sweden

**Keywords:** Placental abruption, Cardiovascular disease, Women, Mortality

## Abstract

Women with preeclamptic pregnancies have increased long-term cardiovascular disease (CVD) mortality. We explored this mortality risk among women with placental abruption, another placental pathology. We used linked Medical Birth Registry and Death Registry data to study CVD mortality among over two million women with a first singleton birth between 1967 and 2002 in Norway and 1973 and 2003 in Sweden. Women were followed through 2009 and 2010, respectively, to ascertain subsequent pregnancies and mortality. Cox regression analysis was used to estimate associations between placental abruption and cardiovascular mortality adjusting for maternal age, education, year of the pregnancy and country. There were 49,944 deaths after an average follow-up of 23 years, of which 5453 were due to CVD. Women with placental abruption in first pregnancy (n = 10,981) had an increased risk of CVD death (hazard ratio 1.8; 95 % confidence interval 1.3, 2.4). Results were essentially unchanged by excluding women with pregestational hypertension, preeclampsia or diabetes. Women with placental abruption in any pregnancy (n = 23,529) also had a 1.8-fold increased risk of CVD mortality (95 % confidence interval 1.5, 2.2) compared with women who never experienced the condition. Our findings provide evidence that placental abruption, like other placental complications of pregnancy, is associated with women’s increased risk of later CVD mortality.

## Introduction

Cardiovascular disease (CVD) is the leading cause of death for women globally [1]. Traditional risk factors and scoring systems underestimate women’s CVD risk resulting in lost opportunities for primary prevention [2, 3]. Some pregnancy complications may be signs of subclinical endothelial dysfunction or vascular disease and thus provide insight into a woman’s long-term risk of developing CVD [4, 5]. It is well established, for example, that women with a history of preeclampsia have an increased risk of CVD later in life [6–8]. A previous study in Norway found an increased risk of cardiovascular disease death among women with preeclampsia in the first pregnancy, particularly among mothers who did not have any additional pregnancies [9].

Placental abruption is an uncommon but potentially serious pregnancy complication involving the premature detachment of the placental lining from the uterus before delivery [10]. Affecting 0.4–1 % of pregnancies, abruption is a major cause of perinatal death and maternal morbidity. Fetal survival depends on the severity of the abruption and gestational age, with perinatal mortality in the range of 9–12 % in high income countries and up to 60 % in settings with inadequate neonatal facilities [10, 11]. The immediate risks to the mother, depending on severity, include obstetric hemorrhage, disseminated intravascular coagulation, renal failure, and in rare cases, death. The etiology of abruption is not well understood. Non-pregnancy associated risk factors are cigarette smoking, alcohol and drug abuse, chronic hypertension and blood-clotting disorders; pregnancy-associated risk factors include multiparity, older age (≥35 years) at pregnancy, abdominal trauma during pregnancy, pregnancy-induced hypertension and preeclampsia [10, 12, 13]. Like preeclampsia, the pathophysiological mechanisms of placental abruption are thought to involve problems in early placental implantation resulting in uteroplacental ischemia and placental insufficiency [14–16]. Little is known about long-term health effects for women who experience abruption. We investigated placental abruption and long-term CVD mortality in a large, population-based registry study in Norway and Sweden.

## Methods

We used linked data from population-based birth and cause-of-death registries to study CVD mortality risk among women giving birth in Norway and Sweden. Linkage of individual-level data was possible due to the unique national identity numbers assigned to all legal residents in each country. The Medical Birth Registry of Norway [17], established in 1967, and the Medical Birth Register of Sweden [18], established in 1973, are based on compulsory notification for live and still births. In Norway, information on all births from 16 completed weeks is recorded. In Sweden, all live births from 22 completed weeks are included in the Birth Register. Before 30 June 2008, stillbirths were included from 28 completed weeks. After that date, stillbirths were included from 22 completed weeks. The registries include prospectively collected data on maternal characteristics and medical history, complications during pregnancy, labor and delivery and conditions of the newborn. Data were recorded in a standard manner by attending midwives and physicians during prenatal visits and at delivery and hospital discharge. Medical conditions, including placental abruption and other pregnancy complications, were diagnosed by physicians and coded using the International Classification of Diseases (ICD) (8th revision for years 1967–1998 in Norway and 1973–1986 in Sweden; 9th revision for years 1987–1996 in Sweden; and 10th revision for years 1999–2009 in Norway and 1997–2010 in Sweden). Placental abruption was defined using ICD-8 codes 632.1 and 651.4, ICD-9 code 641C, and ICD-10 code O45. Mother’s (and in Norway, father’s) lifetime years of formal education (as of 2009 in both countries) were obtained through linkage to population-based education registers in each country.

**Fig. 1:**
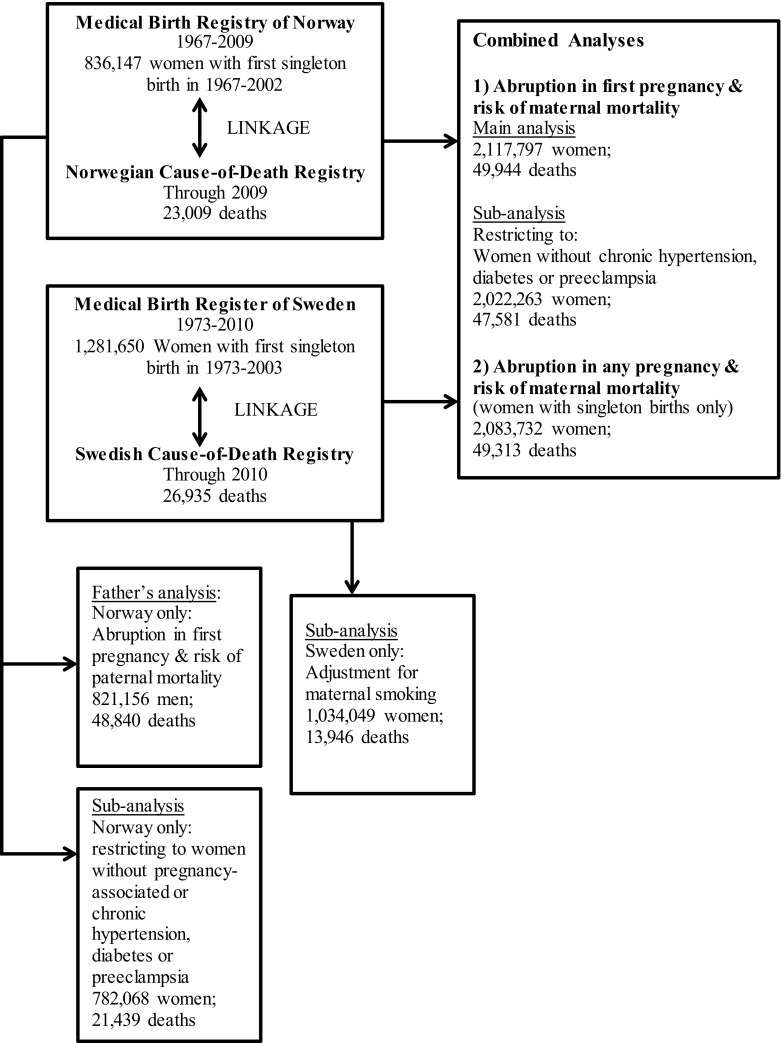
Data sources and numbers of study subjects

Each woman’s birth history was obtained by linking births for the period 1967–2009 in Norway and 1973–2010 in Sweden using the mother’s unique national identification number (Fig. 1). We restricted the study group to women with a first singleton birth between 1967 and 2002 in Norway (n = 836,147) and between 1973 and 2003 in Sweden (n = 1,281,650) in order to be able to identify women who had second births within 7 years after the first birth. (In Norway, 95 % of women with two or more births had their second within 7 years [9].) Maternal (and in Norway, paternal) deaths were identified by data linkage to the respective national Cause-of-Death Registry through December 2009 for Norway and December 2010 for Sweden. Loss of follow-up due to emigration was low (<1 %). In addition to total mortality, we analyzed deaths due to diseases of the circulatory system (ICD-8 and ICD-9 codes 390-459; ICD-10 codes I00-I99). This category was further divided into deaths from ischemic heart disease (ICD-8 and ICD-9 codes 410-414; ICD-10 codes I20-I25), cerebrovascular disease (ICD-8 and ICD-9 codes 430-438 and ICD-10 codes I60-I69), and other circulatory system diseases (all remaining circulatory system codes). Deaths from ischemic heart disease and cerebrovascular disease were combined into one category referred to as “CVD deaths” for our main analyses. We also examined non-CVD deaths (all those not included in the circulatory disease definition above).

We conducted two main sets of analyses. First, we examined placental abruption in the first pregnancy and maternal mortality. We analyzed data from Norway and Sweden separately (n = 836,147 and n = 1,281,650, respectively) and then pooled the data in combined analyses (n = 2,117,797). We used Cox proportional-hazards regression to estimate hazard ratios (HRs) and 95 % confidence intervals (CIs). To enable full adjustment for the effect of age on mortality risk, we used attained age as the primary time scale with women having delayed entry into risk sets (left-censored) at the year of their first births [19, 20]. Women were followed until death or until censored at the cutoff year of 2009 in Norway and 2010 in Sweden. Models were adjusted for maternal age at first birth (<20, 20–24, 25–29, 30–34, 35–39, 40+ years), lifetime years of maternal education (≤9, 10–12, 13–14, 15+ years), and year of first birth (linear term), and, in analyses of combined data, country.

To remove the possible effects of underlying chronic disease or other pregnancy conditions on the association between abruption and maternal mortality, we conducted analyses restricted to women without the following conditions before or during their first pregnancies: chronic hypertension, chronic or gestational diabetes mellitus, preeclampsia, and pregnancy-induced hypertension (available in Norway only). To assess possible differences in the associations by number of births and gestational age, we constructed categorical variables combining the number of births (one vs. two or more) and pregnancy outcome (term and preterm with and without placental abruption). Preterm was defined as delivery in weeks 22 through 36. We evaluated the statistical significance of interactions by creating cross-product terms between abruption and the modifier of interest and using the likelihood ratio test to compare the fit of models with and without the interaction term.

In our second set of analyses, we examined mortality risk in relation to placental abruption in any pregnancy, the total number of occurrences of abruption, and whether or not the woman also had preeclampsia in any of her pregnancies. These analyses were conducted using combined data from Norway and Sweden and were restricted to women with singleton births only n = 2,083,732. We used Cox proportional-hazards regression with attained age as the primary time scale; women had delayed entry into risk sets (left-censored) at the year of their most recent birth because we were assessing exposures across reproductive history. The adjustment variables and follow up period were the same as the first series of analyses described above.

We used subsets of data available only in Norway or Sweden to further assess and adjust for possible confounding. Using information on 821,156 fathers in Norway, we examined abruption in first pregnancy and father’s mortality risk. The objective was to assess the possible role of confounding by unmeasured social factors. Any association of father’s mortality with abruption would presumably be unrelated to the physiologic effects of pregnancy. We used the same selection criteria and analytic strategy as for mothers, with father’s attained age as the underlying time variable in the Cox proportional hazards models, left-censoring at the year of first delivery, and adjustment for father’s age at the birth (<20, 20–24, 25–29, 30–34, 35–39, 40+ years), year of first birth (linear term), and father’s education (9 categories).

In Sweden, information on maternal smoking was ascertained by midwives at the time of the first prenatal care visit (occurring before the 15th week of gestation in over 95 % of the pregnancies) and was available in the Medical Birth Register from 1983-onward. For our analysis of mortality risk, we were interested in women’s smoking status and not the level of smoking in any particular pregnancy. To estimate this, we grouped women as non-smokers (i.e., non-daily smokers) and smokers (i.e., daily smokers) at the first prenatal visit. To maximize the use of the available data, we examined smoking across all of each woman’s pregnancies and categorized women as ever or never smokers, thus avoiding missing data for the women with first pregnancies before 1983 but who had later pregnancies with smoking information. Among the 1,034,049 women with smoking data using this strategy, we assessed possible confounding by comparing estimates from models including and excluding the smoking variable in analyses examining abruption in any pregnancy and risk of maternal mortality.

## Results

### Placental abruption in first pregnancy

Among the 2,117, 797 women who had first deliveries during the study period, 10,981 (0.5 %) had placental abruption (Table 1). The mean follow up time was 25 years in Norway, 22 years in Sweden, and 23 years (range 1–42 years) in the combined material with a total of 48,786,382 person-years at risk. There were 5453 CVD deaths out of a total of 49,994 deaths during the follow up period (22,885 in Norway and 26,760 in Sweden). The median age at death was 48 years overall and 51 years for CVD deaths.

**Table 1:** Maternal and pregnancy characteristics by occurrence of abruption in first pregnancy among women with singleton first births in Norway 1967–2002 and Sweden 1973–2003

Maternal and pregnancy characteristics	Abruption in first pregnancy N = 10,981		No abruption in first pregnancy N = 2,106,816	
	Number	%	Number	%
Age at first pregnancy (years)				
<20	1170	10.7	218,846	10.4
20–24	3849	35.1	787,053	37.4
25–29	3537	32.2	719,829	34.2
30–34	1755	16.0	292,614	13.9
35–39	567	5.2	76,468	3.6
40+	103	0.9	12,003	0.6
Missing^a^	0		3	
Education (years)				
<10	2058	19.1	327,771	15.8
10–12	5146	47.7	975,121	47.1
13–14	1030	9.6	221,670	10.7
15+	2547	23.6	547,661	26.4
Missing^a^	200		34,593	
Place of birth				
Native to Norway or Sweden	9773	89.7	1,862,732	88.9
Other Nordic	402	3.7	77,169	3.7
Other	726	6.7	156,138	7.5
Missing^a^	80		10,777	
Parity by end of follow-up				
One child	2366	21.6	370,494	17.6
Two or more	8615	78.4	1,736,322	82.4
Pre-gestational hypertension^b^	43	0.4	4041	0.2
Pre-gestational diabetes^b^	45	0.4	5223	0.4
Preterm delivery	5263	49.8	122,544	6.0
Missing^a^	404		50,170	
Gestational diabetes^b^	33	0.3	6234	0.3
Preeclampsia^b^	1106	10.1	80,868	3.8
Pregnancy-related hypertension^b,c^	118	2.5	14,266	1.7
Smoked during any pregnancy^d^	1792	34.7	257,450	24.6
Missing	1087		229,132	

^a^Missing values <4 %

^b^Status during first pregnancy

^c^Norway only

^d^Sweden only, women with singleton births only, smoking information available 1983-onward, 18 % missing values

Associations between abruption in first pregnancy and mortality outcomes were generally similar for Norway and Sweden (Table 2); results of the combined analyses are reported here. Compared with women who did not experience placental abruption in first pregnancy, those who experienced placental abruption had an increased risk of CVD mortality (HR 1.8; 95 % CI 1.3, 2.4). Risks were similar for the sub-categories of ischemic heart disease (HR 2.0; 1.4, 2.9) and cerebrovascular disease (HR 1.6; 1.0, 2.4). Women with abruption also had a small increased risk of dying from non-CVD disease (HR 1.2; 1.0, 1.3). Increased risk of CVD mortality persisted after excluding women with chronic hypertension, chronic or gestational diabetes, preeclampsia, and pregnancy-related hypertension (Table 3).

**Table 2:** Placental abruption in first pregnancy and long-term mortality risk by cause of death among women with singleton first births in Norway 1967–2002 and Sweden 1973–2003

Cause of death	Norway			Sweden			Combined		
	Total	Deaths	Adjusted hazard ratio (95 % confidence interval)^a^	Total	Deaths	Adjusted hazard ratio (95 % confidence interval)^a^	Total	Deaths	Adjusted hazard ratio (95 % confidence interval)^a^
Total circulatory disease (ischemic, cerebrovascular, other)									
Placental abruption									
No	831,415	3848	1.00 (Referent)	1,275,401	5371	1.0 (Referent)	2,106,816	9219	1.0 (Referent)
Yes	4732	43	1.8 (1.3, 2.4)	6249	46	1.7 (1.2, 2.3)	10,981	89	1.7 (1.4, 2.2)
Sub-categories of circulatory disease									
Cardiovascular (ischemic, cerebrovascular)									
Placental abruption									
No	831,415	2525	1.00 (Referent)	1,275,401	2873	1.0 (Referent)	2,106,816	5398	1.0 (Referent)
Yes	4732	31	1.9 (1.3, 2.8)	6249	24	1.6 (1.0, 2.5)	10,981	55	1.8 (1.3, 2.4)
Ischemic heart disease									
Placental abruption									
No	831,415	1298	1.0 (Referent)	1,275,401	1417	1.0 (Referent)	2,106,816	2715	1.0 (Referent)
Yes	4732	14	1.8 (1.0, 3.0)	6249	17	2.3 (1.3, 3.8)	10,981	31	2.0 (1.4, 2.9)
Cerebrovascular disease									
Placental abruption									
No	831,415	1270	1.00 (Referent)	1,275,401	1532	1.0 (Referent)	2,106,816	2802	1.0 (Referent)
Yes	4732	17	2.0 (1.2, 3.4)	6249	8	1.0 (0.4, 2.2)	10,981	25	1.6 (1.0, 2.4)
Other circulatory disease									
Placental abruption									
No	831,415	1323	1.00 (Referent)	1,275,401	2498	1.0 (Referent)	2,106,816	3821	1.0 (Referent)
Yes	4732	12	1.5 (0.9, 2.7)	6249	22	1.8 (1.1, 2.8)	10,981	34	1.7 (1.2, 2.4)
Non-circulatory disease									
Placental abruption									
No	831,415	18,983	1.00 (Referent)	1,275,401	21,381	1.0 (Referent)	2,106,816	40,364	1.0 (Referent)
Yes	4732	135	1.2 (1.0, 1.4)	6249	137	1.2 (1.0, 1.4)	10,981	272	1.2 (1.0, 1.3)
Total mortality									
Placental abruption									
No	831,415	22,831	1.0 (Referent)	1,275,401	26,752	1.0 (Referent)	2,106,816	49,583	1.0 (Referent)
Yes	4732	178	1.3 (1.1, 1.5)	6249	183	1.3 (1.1, 1.5)	10,981	361	1.3 (1.1, 1.4)

^a^Adjusted for mother’s age at first birth, education (4 levels), and birth year of child; combined estimates further adjusted for country

**Table 3:** Placental abruption in first pregnancy and long-term mortality risk among women with singleton first births in Norway 1967–2002 and Sweden 1973–2003

Condition of first pregnancy	Number of women	Cardiovascular disease deaths		Non-cardiovascular disease deaths^a^		Total deaths	
		Number	Adjusted hazard ratio (95 % confidence interval)^b^	Number	Adjusted hazard ratio (95 % confidence interval)^b^	Number	Adjusted hazard ratio (95 % confidence interval)^b^
Main analysis							
Placental abruption							
No	2,106,816	5398	1.0 (Referent)	40,572	1.0 (Referent)	49,583	1.0 (Referent)
Yes	10,981	55	1.8 (1.3, 2.4)	273	1.2 (1.0, 1.3)	361	1.3 (1.1, 1.4)
Sub-analyses							
Excluding women with chronic hypertension, pre-pregnancy or gestational diabetes, preeclampsia							
Placental abruption							
No	2,012,488	4941	1.0 (Referent)	38,757	1.0 (Referent)	47,264	1.0 (Referent)
Yes	9775	45	1.7 (1.2, 2.3)	244	1.2 (1.0, 1.3)	317	1.3 (1.1, 1.4)
Norway only: excluding women with the above conditions plus pregnancy-related hypertension							
Placental abruption							
No	777,977	2212	1.0 (Referent)	17,868	1.0 (Referent)	21,290	1.0 (Referent)
Yes	4091	23	1.8 (1.2, 2.8)	117	1.2 (1.0, 1.4)	149	1.3 (1.1, 1.5)

^a^Excluding all circulatory conditions

^b^Adjusted for mother’s age at first birth, mother’s education, birth year of child, and in analyses of combined data, country

For women with two or more pregnancies, abruption in the first pregnancy was associated with an increased risk of CVD mortality (HR 2.1; 1.5, 2.9) (Table 4). For women with one lifetime pregnancy, the increased risk of CVD mortality associated with abruption (HR 2.6; 1.5, 4.3) was only marginally increased above the CVD mortality risk associated with having only one pregnancy (HR 1.9; 1.8, 2.1) (reference group women with two or more pregnancies and no abruption in first pregnancy). The interaction between number of pregnancies and abruption for CVD mortality was not statistically persuasive (*p* value = 0.16). The association between abruption and CVD mortality was present mainly in women who had preterm deliveries (HR 2.3; 1.6, 3.3 for abruption in preterm; HR 1.3; 0.8, 2.2 for abruption in term), however the interaction between preterm delivery and abruption for CVD mortality was not statistically persuasive (*p* value = 0.86).

**Table 4:** Placental abruption in first pregnancy and long-term mortality risk by number of pregnancies among women with singleton first births in Norway 1967–2002 and Sweden 1973–2003

Condition of first pregnancy	Number of women	Cardiovascular disease deaths		Non-cardiovascular disease deaths^a^		Total deaths	
		Number	Adjusted hazard ratio (95 % confidence interval)^b^	Number	Adjusted hazard ratio (95 % confidence interval)^b^	Number	Adjusted hazard ratio (95 % confidence interval)^b^
Placental abruption by no. lifetime pregnancies							
No abruption—2+ pregnancies	1,736,322	3429	1.0 (Referent)	27,529	1.0 (Referent)	33,329	1.0 (Referent)
Abruption—2+ pregnancies	8615	35	2.1 (1.5, 2.9)	172	1.2 (1.0, 1.4)	227	1.3 (1.1, 1.5)
No abruption—1 pregnancy	370,494	1969	1.9 (1.8, 2.1)	12,835	1.7 (1.7, 1.7)	16,254	1.7 (1.7, 1.8)
Abruption—1 pregnancy	2366	20	2.6 (1.5, 4.3)	100	2.0 (1.6, 2.5)	134	2.2 (1.8, 2.6)
Interaction abruption × no. of pregnancies			*p* value = 0.16		*p* value = 0.97		*p* value = 0.84
Placental abruption by preterm							
No abruption—term	1,934,102	4632	1.0 (Referent)	36,263	1.0 (Referent)	44,230	1.0 (Referent)
Abruption—term	5314	21	1.3 (0.8, 2.2)	122	1.1 (0.9, 1.3)	161	1.2 (1.0, 1.4)
No abruption—preterm	122,544	600	1.8 (1.7, 2.0)	2968	1.2 (1.2, 1.3)	3953	1.3 (1.3, 1.4)
Abruption—preterm	5263	30	2.3 (1.6, 3.3)	138	1.3 (1.1, 1.5)	184	1.4 (1.2, 1.6)
Interaction abruption × preterm			*p* value = 0.86		*p* value = 0.64		*p* value = 0.29

^a^Excluding all circulatory conditions

^b^Adjusted for mother’s age at first birth, mother’s education, birth year of child and country

### Placental abruption in any pregnancy

In analyses of placental abruption in any pregnancy, the mean follow up was 23 years (range 1–42 years) among 2,083,732 women, contributing to 48,018,129 person years at risk. A total of 23,529 women or 1.1 % of the study population had placental abruption in any pregnancy. There were 5385 CVD deaths out of a total of 49,313 deaths during the follow up period. Compared with women who never experienced placental abruption, women who had placental abruption in at least one pregnancy had a 1.8-fold increased risk of CVD mortality (Table 5). The HR was slightly higher among the 782 women who had abruption in 2 or more pregnancies (HR 2.2; 0.8, 6.0) based on only 5 CVD deaths in the exposed group. Women who had abruption in 2 or more pregnancies also had an increased risk of dying from a non-CVD cause (HR 1.6; 1.1, 2.5). In conjoint analyses of placental abruption and preeclampsia in any pregnancy, there were increased risks of CVD mortality among women who had placental abruption only (HR 1.8; 1.4, 2.2), preeclampsia only (HR 1.9; 1.7, 2.1), and women experiencing both conditions in either the same or different pregnancies (HR 3.0; 1.9, 4.8) compared with women who had experienced neither.

**Table 5:** Placental abruption in any pregnancy and long-term mortality risk among women with first singleton births in Norway 1967–2002 and Sweden 1973–2003

	Number	Cardiovascular disease deaths		Non-cardiovascular disease deaths^a^		Total deaths	
		Number	Adjusted hazard ratio (95 % confidence interval)^b^	Number	Adjusted hazard ratio (95 % confidence interval)^b^	Number	Adjusted hazard ratio (95 % confidence interval)^b^
Placental abruption							
Never	2,060,203	5272	1.0 (Referent)	39,557	1.0 (Referent)	48,573	1.0 (Referent)
Ever	23,529	113	1.8 (1.5, 2.2)	562	1.2 (1.1, 1.3)	740	1.3 (1.2, 1.4)
No. of times							
Never	2,060,203	5272	1.0 (Referent)	39,557	1.0 (Referent)	48,573	1.0 (Referent)
1	22,747	108	1.8 (1.5, 2.2)	535	1.2 (1.1, 1.3)	706	1.3 (1.2, 1.4)
2+	782	5	2.2 (0.8, 6.0)	27	1.6 (1.1, 2.5)	34	1.7 (1.2, 2.5)
Placental abruption and preeclampsia/eclampsia							
Never had either	1,950,617	4790	1.0 (Referent)	37,751	1.0 (Referent)	46,026	1.0 (Referent)
Placental abruption only	20,701	95	1.8 (1.4, 2.2)	505	1.2 (1.1, 1.3)	654	1.3 (1.2, 1.4)
Preeclampsia only	109,586	482	1.9 (1.7, 2.1)	1806	0.9 (0.9, 1.0)	2547	1.1 (1.0, 1.1)
Both	2828	18	3.0 (1.9, 4.8)	57	1.1 (0.8, 1.4)	86	1.3 (1.1, 1.7)

Excluding women with multiple gestations in any pregnancy

^a^Excluding all circulatory conditions

^b^Adjusted for mother’s age at first birth, mother’s education, birth year of child and country

### Father’s analysis

In the Norwegian data, the association between placental abruption in the first delivery and father’s CVD mortality was small and did not reach statistical significance (HR 1.2; 0.9, 1.5).

### Adjustment for maternal smoking

In the subset of Swedish data with available smoking information from 1983-onward, maternal smoking was associated with both non-CVD mortality (HR 1.7; 1.7, 1.8) and CVD mortality (HR 2.9; 2.5, 3.3). Without adjustment for smoking, the association between abruption in any pregnancy and risk of maternal CVD mortality was HR = 2.0 (1.3, 2.3) after adjusting for maternal age, education and year of first birth. Further adjustment for maternal smoking attenuated this association (HR 1.7; 1.2, 2.5).

## Discussion

In this large, population-based study, women with placental abruption in the first or a later pregnancy had a 1.8-fold increased risk of CVD mortality later in life. The magnitude of the association is similar to that of current smoking and CVD mortality in women [21, 22]. This study adds to the growing evidence that pregnancy complications may predict later CVD and help target women for primary prevention [23]. It is unclear whether the metabolic, inflammatory and hemodynamic demands of pregnancy unmask underlying phenotypic susceptibility to CVD expressed through pregnancy complications or if the complicated pregnancy itself induces vascular damage or inflammatory or other responses that increase CVD risk [24]. Following a complicated pregnancy, postpartum referral by obstetricians to primary-care physicians or cardiologists for monitoring and controlling CVD risk factors would allow women of child-bearing age to begin preventive efforts early in life and reduce the risk of CVD. The American Heart Association recently incorporated history of preeclampsia, gestational diabetes, and pregnancy-induced hypertension into its CVD risk-assessment guidelines for women [25]. Placental abruption should be considered for inclusion as an additional risk factor.

There was a suggestion in our data of a stronger effect of abruption among women who had two or more pregnancies and those with preterm deliveries. However, these interactions lacked statistical strength, which limits our ability to interpret them. Women with only one pregnancy already have a strongly increased risk of CVD mortality (as has been reported earlier [9]), which may make the further influence of abruption difficult to detect. Vaginal bleeding caused by placental abruption is associated with a high risk of preterm delivery [26]. The occurrence of abruption may necessitate or provoke the delivery of the infant early in gestation [27]. In our study, about half of the women with abruption in first pregnancy had pregnancies ending in preterm delivery. Preterm delivery itself is associated with increased risk of maternal CVD [28, 29], suggesting that it may be in the causal pathway for the association between abruption and CVD mortality.

The results of the father’s analysis provide some assurance that our main findings in mothers were not primarily due to unmeasured confounding of lifestyle variables. There was evidence of a small increased risk of paternal CVD mortality for first-pregnancy abruption (although confidence intervals included one), suggesting that our findings may have a small degree of confounding by unmeasured social factors or CVD risk factors, such as cigarette smoking, that have a high concordance in partners [30, 31]. In our sub-analysis of Swedish data, adjustment for maternal smoking left most of the association between abruption and maternal CVD mortality, although our smoking data were limited to information collected at the first prenatal care visit and residual confounding is still possible. To the extent that smokers were misclassified in our sub-analysis, the association between abruption and risk of CVD mortality may be overestimated.

The increased risk for non-CVD mortality among women who experienced abruption in two or more pregnancies is notable. Among the 34 women with recurrent abruption who died of non-CVD causes, 18 % had drug or alcohol-related deaths compared with only 6 % of the deaths among women without abruption. Injury-related deaths, including suicide, were also increased (29 vs. 16 %), but not cancer deaths (32 vs. 52 %). This suggests that the increased risk of non-CVD mortality among women with recurrent abruption was due, at least in part, to drug or alcohol abuse, factors also related to increased risk of abruption.

The prevalence of abruption in our study is similar to that of other epidemiologic studies relying on clinical diagnosis. Placental abruption has not been validated in the Norway Birth Registry, but a 1986 validation study in Sweden found good correspondence between the diagnosis of abruption as reported by ICD codes in the birth registry and the description of clinical abruption documented in individual delivery records [32]. In the United States, two validation studies comparing state birth certificates with medical records found >99 % specificity and moderate sensitivity for abruption, as would be expected for a rare condition [33, 34]. This suggests that studies using vital statistics registries are subject to under-ascertainment of rare conditions such as abruption but have negligible numbers of false positives. With the assumption that exposure misclassification is non-differential in regard to outcome, which seems reasonable in our study because the collection of exposure data in registries predated the outcome, any bias produced by exposure misclassification would tend to be toward the null.

The strengths of this study include the use of population-based registry data from two Nordic countries, which enabled a large study size, prospective data collection, the study of complete reproductive history, and nearly complete ascertainment of mortality. Limitations of the study include a lack of detailed information on underlying cardiovascular risk factors before and after pregnancy. We also lacked information on the severity of the abruption and could not differentiate between mild cases and the more serious manifestations. Even in our study of over 2 million women, we had small numbers of women for some of the exposures of interest, particularly recurrent abruption, which limited our statistical power in some analyses. Although we lacked information on maternal smoking for the full study, we were able to use available Swedish data to adjust for smoking in sub-analyses. However, there is likely some misclassification of smoking in these analyses; for example, women who smoked either before or after their pregnancies but abstained during pregnancy would have been misclassified as non-smokers in our study. Even with an average of 23 years of follow-up, the women in this study were relatively young at the end of follow up. The CVD deaths that occurred at a relatively young age among women with abruption may reflect the most severe cases of the condition, and therefore the associations between abruption and risk of CVD mortality may attenuate as the follow up period increases over time, as observed in a study of preeclampsia [9].

Two previous studies examined placental abruption and long-term maternal CVD mortality. A large registry-based study in Denmark of 782,287 women with singleton deliveries 1978–2007 (7684 with abruption) and a median follow-up of 14.8 years found a weakly increased risk of CVD mortality for women with abruption (HR 1.23; 0.78, 1.93) [35]. Their CVD endpoint differed from ours; along with a CVD cause of death stated in the Cause of Death Registry, they also included a first clinical diagnosis of CVD (e.g., de novo hypertension) within 1 week prior to death. If their broader definition resulted in misclassification of non-CVD deaths as CVD deaths, then the resulting bias would tend to be towards the null. An Israeli study examined CVD mortality among 47,585 women (653 with abruption) who gave birth in a large medical center during 1988–1999 with a median follow up of 13.8 years [36]. They found an increased risk of CVD mortality for women with abruption (HR 4.3; 1.1–18.6). This hospital-based study lacked information on CVD mortality outside of the medical center and the small numbers of outcomes (only 6 CVD deaths among exposed women) contributed to imprecise estimates. Our use of registry data from two countries allowed population-based ascertainment of CVD deaths and provided a larger sample of women and longer follow-up time than these previous studies.

In summary, we found evidence that placental abruption, like other placental complications of pregnancy, is associated with women’s increased risk of later CVD mortality. Pregnancy complications occur at an early phase of life when targeted prevention may allow women to avoid CVD through lifestyle changes or preventive medicine. It is currently unknown whether traditional prevention measures will be effective in reducing long-term CVD risk in this population of women [37]. The feasibility of changing the course of women’s CVD risk after placental complications of pregnancy should be explored.
